# How SARS-CoV-2 and Other Viruses Build an Invasion Route to Hijack the Host Nucleocytoplasmic Trafficking System

**DOI:** 10.3390/cells10061424

**Published:** 2021-06-07

**Authors:** Elma Sakinatus Sajidah, Keesiang Lim, Richard W. Wong

**Affiliations:** 1 Division of Nano Life Science in the Graduate School of Frontier Science Initiative, Kanazawa University, Kanazawa 920-1192, Japan; elmasajidah@stu.kanazawa-u.ac.jp; 2 WPI-Nano Life Science Institute, Kanazawa University, Kanazawa 920-1192, Japan; 3 Cell-Bionomics Research Unit, Institute for Frontier Science Initiative, Kanazawa University, Kanazawa 920-1192, Japan

**Keywords:** viral nuclear import, viral nuclear export, nucleoporins, importins, exportin, SARS-CoV-2, Ebola virus, Dengue virus, Human Immunodeficiency virus, Influenza A, Human Papillomavirus, Hepatitis B virus, Epstein-Barr virus, antiviral drug, nuclear transport inhibitor, clinical trial, HS-AFM

## Abstract

The host nucleocytoplasmic trafficking system is often hijacked by viruses to accomplish their replication and to suppress the host immune response. Viruses encode many factors that interact with the host nuclear transport receptors (NTRs) and the nucleoporins of the nuclear pore complex (NPC) to access the host nucleus. In this review, we discuss the viral factors and the host factors involved in the nuclear import and export of viral components. As nucleocytoplasmic shuttling is vital for the replication of many viruses, we also review several drugs that target the host nuclear transport machinery and discuss their feasibility for use in antiviral treatment.

## 1. Introduction

Despite advancements in science and technology, humans are still plagued by communicable diseases, and the development and production of effective antiviral drugs and vaccines remains challenging. Pathogenic viruses, one major category of infectious agents, have caused not only substantial morbidity and mortality, but also devastating socioeconomic impacts. Within the past century, there have been two particularly severe pandemics, as follows: the 1918 influenza pandemic and the currently ongoing coronavirus disease 2019 (COVID-19) pandemic.

As part of their lifecycle, many viruses hijack the host transcription and translation machinery while evading the host immune responses [1]. The host nucleoplasm and cytoplasm are segregated by a nuclear envelope (NE), a lipid bilayer embedded with numerous nano-gates known as nuclear pore complexes (NPCs) (reviewed in [2]). Nuclear transport is important for mediating numerous cellular activities, such as cell division [3,4], cell metabolism [5,6], gene regulation (review in [7]), and innate immune response activation [8]. As exploitation of the host nuclear transport is important for viral replication, the mechanisms that are used by viruses to hijack the host nuclear trafficking are potential targets for antiviral drugs; such drugs might halt viral genome transcription, viral protein synthesis, and viral assembly. In this review, we focus on the various mechanisms used by viruses, including severe acute respiratory syndrome coronavirus 2 (SARS-CoV-2), to hijack the host nucleocytoplasmic trafficking machinery. We describe various viral factors, and their target host factors, including importins and nucleoporins (Nups). Finally, we discuss the feasibility of using drugs that target the host nuclear transport machinery as antiviral therapies.

## 2. Fundamentals of Nucleocytoplasmic Trafficking

NPCs are mega-Dalton-sized protein complexes that consist of about thirty different types of Nup. Cryo-electron microscopy and tomography observations indicate that NPCs are formed by an eight-fold symmetric central scaffold, eight cytoplasmic filaments, and eight nucleoplasmic filaments (nuclear basket), with the pores presenting rotational symmetry [9,10,11]. Phenylalanine-glycine (FG) repeats are found in many Nups, and the dynamic hydrophobic interactions among the FG-repeats of Nups in the central scaffold create a cohesive meshwork that can make the NPC become a selective channel [12,13]. Several models to describe the FG-containing Nup interactions, including the hydrogel model [14], virtual gating or polymer brush model [13], reduction in dimensionality model (reviewed in [15]), and forest model [16], have been put forward. Recently, we successfully observed the dynamic behavior of Nup FGs by using high-speed atomic force microscopy (HS-AFM) and proposed a new model of FG-containing Nup interactions called the coweb FG-network model [17]. The functional NPC pore diameter was initially reported as being approximately 26 nm [18], but the value was later corrected to approximately 40 nm [19]. Small molecules (<40–60 kDa) pass freely through NPCs via passive diffusion. Conversely, large molecules, such as proteins, require receptor-mediated nuclear transport to move against a concentration gradient [19].

Receptor-mediated nuclear transport involves cargo proteins, nuclear transport receptors (NTRs), and the guanosine triphosphate-binding nuclear protein Ran (RanGTP) [2]. The types of NTRs required for transport differ depending on the traffic directionality. For nuclear import, selective cargo is recognized by specific NTRs via its nuclear localization signal (NLS) sites [2]. According to reviews authored by Pumroy et al. [20] and Mosammaparast et al. [21], several nuclear import pathways are available, depending on the cargo type. The best understood import pathway is the classical import pathway. It utilizes the heterodimeric importin-α/β1 transport receptor, in which importin-α works as an adaptor protein, bringing its cargo in concert with importin-β to translocate it into the nucleus. There are seven importin-α isoforms expressed in humans, and they can be grouped into the following three subfamilies: α1, α2, and α3 (see Table 1). The conserved N-terminal autoinhibitory importin-β-binding (IBB) domain and the C-terminal Armadillo (Arm) repeats (the NLS-binding sites) of α-importins are important for the nuclear import of the NLS-bearing cargo [22,23,24].

Most of the identified NLS motifs consist of basic amino acids, such as lysine and arginine, and they are collectively named the classical NLSs (cNLSs). These NLSs are divided into the following two groups: monopartite cNLSs, which have a single stretch of amino acids (e.g., the NLS of the SV40 large T antigen), [25] and bipartite cNLSs, which have two stretches of amino acids separated by a linker region (e.g., the NLS of nucleoplasmin) [26]. There are many cargoes whose NLSs are still unknown. First, the cNLS-bearing cargo binds to the Arm repeats of importin-α [24], and then importin-β binds to the IBB domain of importin-α [27] to form a ternary complex. The IBB domain can be considered as an NLS for α-importins (i.e., a ubiquitous adaptor protein) as well as for other adaptor proteins that carry specific cargo (see Table 1). Once a ternary complex is formed by the cNLS-bearing cargo, importin-α, and importin-β1, the complex docks onto an NPC; subsequently, importin-β1–Nup FG interactions translocate the complex into the nucleus [28].

RanGTP is predominant in the nucleus, whereas the guanosine diphosphate-binding Ran (RanGDP) is abundant in the cytoplasm (reviewed in [29]). This RanGTP/RanGDP gradient is important for the dissociation of the cNLS-bearing cargo–importin-α/β1 ternary complex and the shuttling of importins back to the cytoplasm for the next functional cycle. RanGTP has a high affinity for importin-β1 [30], and the binding of RanGTP to importin-β1 dissociates the ternary complex, thus releasing the cargo in the nucleus [31,32,33]. The RanGTP–importin-β1 complex is then exported to the cytoplasmic site of an NPC. Similarly, the nuclear export of importin-α also requires RanGTP along with a soluble transport factor known as the CAS (Cellular Apoptosis Susceptibility gene) protein or exportin-2 (XPO2) [34]. The hydrolysis of RanGTP to RanGDP is mediated by the RanGTPase-activating protein (RanGAP1) [35,36] and the RanGTP-binding protein (RanBP1) [37,38] at the cytoplasmic site of an NPC, which helps to release importin-α and -β1 back to the cytoplasm. RanGDP is then reimported to the nucleus by nuclear transcription factor 2 (NTF2) [39]. RCC1, the major nucleotide exchange factor of Ran, converts RanGDP back to RanGTP [40].

The nuclear export of cargo requires NTRs (exportins) that read the nuclear export signals (NESs) within cargo [22,24,41]. Chromosomal region maintenance 1 (Crm1), more commonly known as exportin 1 (XPO1), is the major export receptor for roughly 1,000 different leucine-rich-NESs-containing cargoes in human cells [26,42]. For a list of other exportins, please refer to Table 1. A Crm1-mediated nuclear export starts with the formation of a Ran-binding protein 3 (RanBP3)–Crm1–RanGTP–NES-bearing cargo export complex. RanBP3 binds to Crm1 via its FG domains [43]. RanBP3 increases the affinity of the RanBP3–Crm1 complex for RanGTP and the NES-bearing cargo [43]. The quaternary export complex translocates through the NPCs by interacting with Nups and then docks at its terminal docking site, the cytoplasmic Nup214–Nup88 complex [44]. In most cases, RanGAP1 is not soluble but tethered to the NPCs via RanBP2. Cytoplasmic RanBP1 and RanGAP1 mediate RanGTP hydrolysis to disassemble the export complex [45]. A study reported that Crm1 interacts with NPCs in a Nup358/RanBP2-dependent manner [46]. In addition, in vitro experiments have shown that the isolated Ran-binding domain of Nup358 also induces the dissociation of Crm1-export complexes [47]. Collectively, these studies indicate that Crm1-export complexes dissociate after their interaction with soluble RanBP1 and/or Nup358, together with soluble and/or Nup358-associated RanGAP. Free Crm1 then interacts transiently with Nup358 to re-shuttle back to the nucleus [44]. 

Similar to cargo export, RNA export also requires specific receptors, which depend on the RNA type, in concert with adaptor proteins. These export receptors include the following: Nxf1 or TAP, the main mRNA export receptor [48]; Crm1, for ribosomal (r)RNA [49], small nuclear (sn)RNA [50], and some subsets of messenger (m)RNA [51]; Xpot, for transfer (t)RNA [52,53]; and exportin 5 (XPO5), for micro (mi)RNAs [54]. Several pathways mediate mRNA export, and the Nxf1-dependent pathway is the dominant pathway for exporting bulk mRNAs [55,56,57]. In an Nxf1-dependent mRNA export, mature mRNAs are packaged into messenger ribonucleoprotein particles (mRNPs) that then recruit mRNA export factors (e.g., an Nxf1–Nxt1 heterodimer) [58,59]. The interaction between an Nxf1–Nxt1 heterodimer, Rae1, and Nup98 enables mRNA to be delivered to the cytoplasm [60]. Nxf1 has two binding sites for Nxt1 (amino acid [aa] residues 372–445 [61] and 508–583 [60]) and Rae1 (aa residues 1–60 and 372–445) [60]. Only one Nxf1 binding site is necessary for Nxt1 binding (aa residues 372–445) [61], whereas the Nxf1–Rae1 interaction requires both binding sites [60]. The Nup98-binding site of Nxf1, which is located at the C-terminus of Nxf1 (aa residues 601–619), has the highest affinity for the GLFG-repeat domain of Nup98 [60]. Nup98 binds stably with Rae1, and the Rae1 that is pre-bound to Nup98 will not bind to Nxf1 [60]. Therefore, Nup98 provides a bridging site through which Nxf1 can bind to the adjacent site of Rae1 when both simultaneously interact with Nup98 [60]. This mechanism accomplishes mRNA export in a RanGTP-independent manner. Although Nxf1 has an RNA-binding domain at its C-terminus that can directly bind to the constitutive transport element (CTE) RNA of simian type D retroviruses [62], the Nxf1-Nxt1 heterodimer most frequently requires dedicated export adaptors, including Aly/REF and UAP56 (review in [63]). Likewise, Crm1 does not bind directly to mRNA; instead, it creates complexes with different types of mRNA-binding adaptor proteins, such as HuR [64], Nxf3 [65], and LRPPRC [66], to accomplish mRNA export in a RanGTP-dependent manner. In addition to interacting with mRNA, Crm1 also interacts with the adaptor protein Nmd3 to export the 60S ribosomal subunit [67].

## 3. Mechanisms of the Host Nuclear Transport Machinery Hijacking by Viruses

**Figure 1:**
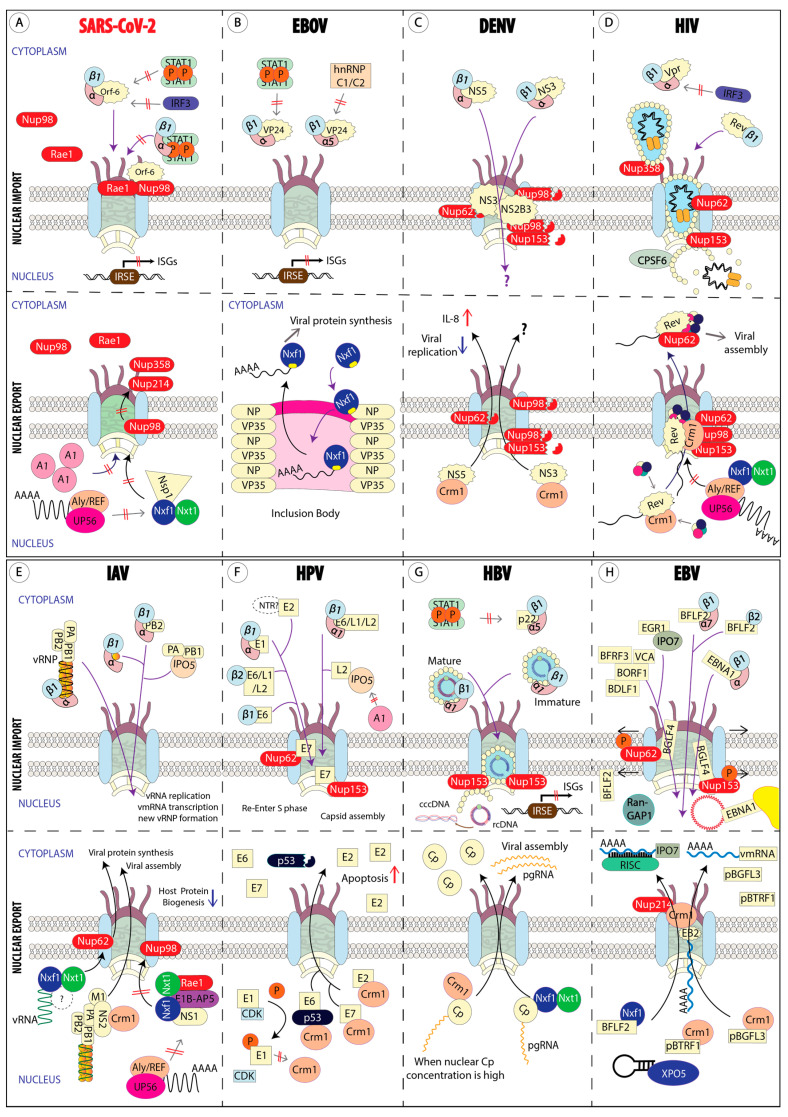
Interaction between viral factors and host factors to hijack host nuclear transport machinery. (**A**) SARS-CoV-2 expresses Orf6 to suppress host antiviral response and Nsp1 to halt host protein biogenesis. (**B**) Ebola virus expresses VP24 to suppress host antiviral response. Ebola virus does not require nuclear export, but it needs the mRNA export factor, Nxf1, to mediate viral mRNA export from the inclusion body. (**C**) Dengue virus NS3 and NS2B3 degrade host Nups. The effect of the nucleocytoplasmic trafficking of both NS5 and NS3 in the host have yet to be elucidated. Nuclear export of Dengue NS5 is associated with elevated IL-8 production and reduced viral replication without a defined mechanism. (**D**) HIV capsids (yellowish balls) interact with host Nups (Nup358, Nup62, and Nup153) and CPSF6, then disassemble at an NPC basket to release PIC. HIV expresses Vpr to suppress host antiviral IFN production by blocking the nuclear translocation of activated IRF3. Nuclear import of HIV Rev is directly mediated by importin-β1. Nuclear Rev protein is needed to form a viral ribonucleoprotein (vRNP) transport complex to export vRNP to the cytoplasm via a Crm1-mediated pathway. (**E**) The Influenza-A virus nucleoprotein (NP, yellowish balls) interacts with importin-α/β1 to enter the nucleus for viral replication. Newly synthesized NPs and RdRp components (PA-PB1 and PB2) hijack host importins to enter the nucleus for new vRNP formation. IAV NS2 facilitates the nuclear export of new vRNP, whereas IAV NS1 blocks host mRNA export by forming an inhibitory complex with Nxf1. (**F**) HPV high-risk E6 and E7 nuclear imports induce carcinogenesis by reprogramming the cell cycle to an S-phase and promote degradation of p53. In addition, HPV viral capsid proteins (L1 and L2) exploit host importins to translocate to the nucleus for capsid assembly. Nuclear export of E2 of high-risk HPV triggers cellular apoptosis. (**G**) Mature HBV capsid interacts with importin-α/β1 and Nup153 to uncoat the capsid in the nuclear basket. Immature HBV capsid remains in the NPC channel after it interacts with Nup153. HBV p22 blocks the nuclear translocation of p-STAT1 to suppress host antiviral response. HBV pgRNA export is mediated either by an Nxf1-Nxt1-dependent or a Crm1-dependent pathway, depending on the nuclear Cp concentration. (**H**) EBV EBNA1 translocates to the nucleus to tether EBV episomal DNA on host chromosome (yellow region). EBV BGLF4 disrupts the NPC structure and promotes nuclear accumulation of RanGAP1 to promote nuclear ingress of viral proteins (VCA, BFRF3, BDLF1, and BORF1) and inhibits host classical importin-α/β1 nuclear import. EBV tegument proteins (pBTRF1 and pBGLF3) bind with Crm1 to translocate to the cytoplasm for viral tegumentation. On the other hand, EBV miRNA is carried to the cytoplasm by host Exportin-5 (XPO5) to suppress importin-7 (IPO7) translation.

The dominant NTRs, including heterodimeric importin-α/β, Crm1, and Nxf1, together with the Nups involved in nuclear transport (Nup358, Nup214, Nup98, and Rae1), are the common targets that viruses hijack for shuttling viral factors between the cytoplasm and the nucleus. The viral replication site determines the purpose of the host nuclear transport subversion by the virus. For example, viruses that replicate in the cytoplasm tend to hijack the host nuclear transport for suppressing the interferon (IFN)-inducing antiviral responses. Conversely, viruses that replicate in the nucleus and assemble their products in the cytoplasm generally exploit the host nuclear transport for much more complicated activities, such as the nuclear import of a viral genome and the nuclear export of a newly synthesized viral genomic RNA to the cytoplasm. Below, we discuss the hijacking mechanisms employed by viruses in various viral families, grouped according to their site of replication, that is, cytoplasm (the *Coronaviridae*, *Filoviridae*, *Flaviviridae*, and *Togaviridae* families) or nucleus (the *Orthomyxoviridae*, *Retroviridae*, *Papillomaviridae*, *Hepadnaviridae*, and *Herpesviridae* families). We summarize these interactions in Table 2 and illustrate them in Figure 1.

### 3.1. Viruses That Replicate in the Cytoplasm

#### 3.1.1. β-Coronaviruses (SARS-CoV-2, SARS-CoV-1, and MERS-CoV)

Coronaviruses (CoVs; members of the *Coronaviridae* family) are enveloped, positive-sense, single-stranded (+ss) RNA viruses. There are the following four genera in the Coronaviridae family: *Alphacoronavirus* (α-CoV), *Betacoronavirus* (β-CoV*)*, *Gammacoronavirus* (γ-CoV*)*, and *Deltacoronavirus* (δ-CoV*)* (reviewed in [68]). Although most human CoVs (HCoVs) are associated with mild upper respiratory diseases and enteric diseases (reviewed in [69]), the recently emerged zoonotic β-CoVs, such as severe respiratory acute syndrome CoV (SARS-CoV) and Middle East respiratory syndrome CoV (MERS-CoV), have caused severe lower respiratory diseases with high mortality (reviewed in [70]). Furthermore, a highly infectious novel β-CoV, SARS-CoV-2, that was first reported in Wuhan, China in 2019 has caused the greatest pandemic in the 21st century [71]. All CoVs, including SARS-CoV-2, replicate in cytosolic double-membrane vesicles (DMVs) [72,73].

A protein–protein interaction (PPI) analysis of the viral factors and the host factors of SARS-CoV-2 revealed that the viral factors NSP9, NSP15, and Orf6 can interact with the host nuclear transport machinery [74]. The associations suggested by these PPI analyses need to be validated by further experiments. To date, only Orf6 has been shown to interrupt the host nucleocytoplasmic trafficking [75,76,77,78] and to induce an aberrant distribution of Nups (Nup98 and Rae1) [76]. Xia et al. found that Orf6 binds to importin-α1 to block the nuclear translocation of IRF3, resulting in an impaired type I IFN production in the HEK293T cell line [78]. Miorin and colleagues reported that type Ⅰ and type Ⅱ IFNs failed to induce the transcription of IFN-stimulated genes (ISGs) in SARS-CoV-2-infected Vero E6 cells owing to an impediment of the STAT1 and STAT2 nuclear translocation by Orf6 [75]. SARS-CoV-1 Orf6 plays a similar role in antagonizing IFN; it binds to importin-α1 and tethers importin-β1 to the endoplasmic reticulum (ER)/Golgi membrane, sequestrating the NTRs from activated STAT1 (pY-STAT1) [79], thus blocking the nuclear translocation of a pY-STAT1 [79,80]. Miorin and colleagues proposed two mechanisms to explain this blockade. First, Orf6 binds to Nup98 via its C-terminal domain to interrupt the docking of cargo–importin-α5/β1 ternary complexes at the NPCs. The Orf6–Nup98 interaction does not affect the Nup98–Rae1 interaction, which suggests that Orf6 binding to Nup98 targets only the nuclear import pathway. Second, Orf6 competes with STAT1 and STAT2 for α-importins (α5 and α1) to block the STAT1/2 nuclear ingress. Nevertheless, these α-importins may not be the direct key players because their overexpression failed to rescue an Orf6-dependent blockade of green fluorescent protein (GFP)-tagged STAT1 nuclear import. Interestingly, Addetia et al. reported that the SARS-CoV-2 Orf6 interactions with Nup98 and Rae1 were much stronger than those of SARS-CoV-1 Orf6, suggesting the strong IFN antagonism triggered by SARS-CoV-2 contributes to the high prevalence of asymptomatic cases of SARS-CoV-2 infection [77].

We found that, along with being able to impair nuclear import, SARS-CoV-2 Orf6 can also block nuclear export [76]. Our results show mislocalizations of Nup98 and Rae1 in Orf6-overexpressing cells [76]. In addition, nuclear accumulation of the nuclear RNA-binding protein hnRNPA1 was observed in Orf6-overexpressing cells. For mRNA export, hnRNPA1 is needed [81,82], and the saturation of hnRNPA1 in the nucleus inhibits mRNA export [81]. These findings, that is, the aberrant localization of Nup98 and Rae1 and the nuclear saturation of hnRNPA1, suggest that the Orf6–Nup98–Rae1 interaction can block mRNA export. Addetia et al. later found that SARS-CoV-2-infected cells exhibited a nuclear mRNA accumulation that could be mediated by Orf6 [77]. Recently, Zhang et al. reported that SARS-CoV-2 non-structural protein 1 (Nsp1) halted the host gene expression by blocking an Nxf1–Nxt1-mediated mRNA export [83]. Mechanistically, Nsp1 did not impair the RNA-binding ability of Nxf1; rather, it interrupted Nxf1 interacting with mRNA export adaptors, including Aly/REF and UAP56. Furthermore, Nsp1 reduced the interactions between Nxf1 and key Nups for nuclear export (Nup358, Nup214, and Nup98). SARS-CoV-1 Nsp1 was previously found to disrupt the localization of Nup93 (from the NE to the nucleoplasm) without affecting the NPC structure [84]. Furthermore, SARS-CoV-1 Nsp1 specifically promotes the cytoplasmic accumulation of the nuclear RNA-binding protein nucleolin with unknown consequences. These findings may indicate a role for SARS-CoV-1 Nsp1 in disrupting the host mRNA export [84].

SARS-CoV-1 Orf9b lacks an NLS and thus is localized mainly in the cytoplasm [85]. However, a small amount of Orf9b enters the nucleus passively, and nuclear Orf9b activates the host caspase-3-mediated apoptosis. As Orf9b degradation occurs in the cytoplasm, the nuclear export of Orf9b can protect the host and sustain viral replication [85]. Orf9b possesses an NES at aa residues 46–54 (LRLGSQLSL) that allows it to bind to Crm1 [85]. MERS-CoV Orf4b possesses an NLS (aa residues 2–38), and its nuclear localization is important for inhibiting IRF3- and IRF7-induced IFN-β production [86]. Canton et al. later demonstrated that MERS-CoV Orf4b has a strong affinity for importin-α3, which blocks the NF-κB p65 subunit from entering the nucleus [87].

#### 3.1.2. Ebola Virus (Zaire Ebolavirus)

Ebola virus (EBOV), a member of the *Filoviridae* family, is a filamentous, enveloped, single-stranded, negative-sense (−ss) RNA virus [88]. At present, there are six recognized EBOV species. The *Zaire* *ebolavirus* species has caused two outbreaks in Africa with 40 and 66% fatality, respectively, [89]. As EBOVs replicate in the cytoplasm, they hijack the host nuclear transport to suppress IFN-mediated antiviral responses. The EBOV VP24 protein interacts with importin-α5, -α6, and -α7 [90], but does not interact with importin-α1, -α3, or -α4 [91]. A more recent study found that EBOV VP24 recognizes a non-classical NLS-binding site in importin-α6 [92]. These interactions allow EBOV to suppress the host IFN signaling by preventing the nuclear translocation of pY-STAT1 [91]. The VP24–importin-α interaction also inhibits IFN-λ production [93]. VP24 can directly bind to the inactive form of STAT1, unphosphorylated STAT1 (U-STAT1) [94]. The nuclear import of U-STAT1 is mediated by Nup153 and Nup214, independently of importins [95]. Within the nucleus, U-STAT1 activates and prolongs the expression of a set of IFN-induced genes that are distinct from those mediated by pY-STAT1 [96]. The VP24–importin-α5 interaction causes a cytoplasmic accumulation of the nuclear protein hnRNP C1/C2 [97]. During mitosis, hnRNP C1/C2 is exported to the cytoplasm for internal ribosomal entry site (IRES)-dependent c-myc translation [98]. Several viruses similarly re-localize hnRNP C1/C2 to the cytoplasm, either for viral replication or IRES-dependent viral protein translation [99,100,101,102], which suggests that VP24-dependent hnRNP C1/C2 cytoplasmic accumulation is essential for EBOV replication [97]. Together, nucleoproteins (NPs) and VP35 form EBOV inclusion bodies (IBs) for viral replication [103]. A study conducted by Gabriel et al. showed that importin-α7 is also involved in IB formation [104]. In addition to creating new copies of EBOV genomic RNA, sub-genomic EBOV RNAs (viral mRNAs) are also produced for viral protein synthesis. The EBOV NP recruits Nxf1 by interacting with the RNA-binding domain of Nxf1. In the presence of mRNA, the binding preference of the Nxf1 RNA-binding domain shifts from NPs to mRNA, and then it delivers mRNA to the cytoplasm for translation [105].

#### 3.1.3. Dengue Virus (DENV) and Zika Virus (ZIKV)

Flaviviruses (members of the *Flaviviridae* family) are small, enveloped +ssRNA viruses that replicate in vesicle packets located at the ER [106,107]. The flavivirus Dengue virus (DENV) causes mosquito-borne dengue fever in tropical and sub-tropical countries. Two nonstructural proteins (NSs), the DENV NS3 helicase and the DENV NS5 RNA-dependent RNA polymerase (RdRp), form a complex to mediate the viral replication [108,109]. NS5 promotes STAT2 degradation to suppress the host IFN-mediated antiviral responses [110]. An early study reported that NS5 has a bipartite NLS composed of an importin-β1-binding site (βNLS) [111] and a heterodimeric importin-α/β1-binding site (cNLS) [112]. NS5 nuclear import is predominantly mediated by importin-α/β1 receptors [112]. However, later studies suggested that these mapped NLSs are not accessible to the host importins [113]. The subcellular localization of NS5 varies among the four DENV serotypes (DENV 1, 2, 3, and 4); the NS5 proteins of DENV2 and DENV3 reside in the nucleus, but the NS5 proteins of DENV1 and DENV4 are located in the cytoplasm [114,115]. A new monopartite NLS of NS5 has been identified at its C-terminus [115]. An in vitro assay revealed that the NS5 proteins of DENV2 and DENV3 have similar and strong affinities for importin-α2, whereas those of DENV1 and DENV4 have weak affinities for importin-α2 [115]. The NES in NS5 allows for Crm1-dependent NS5 nuclear egress, and this egression correlates with elevated IL-8 production and impaired viral replication, the mechanism of which has not yet been defined [116]. An NE abnormality together with deregulated NPC components were found in DENV-infected cells [117]. NS3 and its cofactor, NS2B3, disrupt the NPC integrity by inducing the proteolytic degradation of FG-Nups, including Nup62, Nup153, and Nup98 [117]. Palacios et al. reported that NS3 has a putative NLS and a putative NES. NS3 was localized within the nucleus during the early stages of infection, whereas it remained predominantly in the cytoplasm during the later stages of infection [118].

Another member of the *Flaviviridae* family, Zika virus (ZIKV), has posed serious health concerns owing to the rapid increase in cases of neonatal microcephaly linked to ZIKV-infected mothers in Brazil [119,120]. Similar to DENV, ZIKV also exploits the host NTRs and Nups during infection via its NS3 helicase and NS5 RdRp. A study conducted by De Jesús-González et al. showed that ZIKV NS3, in concert with NS2B3, promotes the proteolytic degradation of several FG-containing Nups, including TPR, Nup153, and Nup98, and also disrupts the NE structure [117]. ZIKV NS5, similar to DENV NS5, has a bipartite NLS (a βNLS and a cNLS), which is recognized by importin-α7 [121]. The nuclear import of ZIKV NS5 protects it from cytoplasmic degradation, and this strategy helps to sustain viral replication in the host cells [122]. The cNLS was initially thought to be the primary site of NTR binding [123], but a later study revealed that both the βNLS and the cNLS are required for NS5 nuclear import [122]. Nuclear NS5 sequesters various α-importins (α1, α3, and α4) in nuclear bodies [123]. Intriguingly, the NS5 accumulated in nuclear bodies was incorporated with STAT1 in a glioblastoma cell line (LN229) but not in a hepatocellular carcinoma cell line (Huh-7), suggesting a role for NS5 in the tissue-specific activation of inflammatory responses [123]. Unlike the NS5 protein in Japanese encephalitis virus (JEV), which competes with the host IRF3 and NF-κB for α-importins (α1, α3, and α4) [124], ZIKV NS5 inhibits the activation of TANK-binding kinase 1 to prevent IRF3 activation [125], a strategy used to inhibit the IFN production by infected cells. Furthermore, ZIKV NS2A promotes the chaperone-mediated autophagy (CMA) of importin-α1 [126], possibly in an effort to suppress the host antiviral response.

#### 3.1.4. Chikungunya Virus (CHIKV)

Chikungunya virus (CHIKV), a member of the *Togaviridae* family, is an enveloped +ssRNA virus that replicates in the host cytoplasm [127]. A CHIKV infection is associated with chronic inflammatory arthritis and other musculoskeletal diseases [128]. The CHIKV capsid protein (CP) has been reported to have two NESs and one NLS [129,130]. Thomas and colleagues demonstrated that the CP bound specifically to the C-terminal NLS-binding site of importin-α3 for its nuclear translocation [129]. The CP binds to Crm1 via its Crm1-mediated NES, which was mapped to a leucine-rich region between aa residues 143 and 155 [129]. Thus, importin-α3 and Crm1 are the host factors targeted by CHIKV in its disruption of the host nucleocytoplasmic trafficking [129]. A mutation of the CHIKV CP’s NES near the N-terminus (aa residues 44–53) caused the retention of viral CPs in the nucleus and also blocked the host nuclear import system for unknown reasons [130]. CHIKV nsP2 inhibits the host IFN-induced antiviral response [131,132,133,134]. Although nsP2 lacks an NLS (reviewed in [135]), the nuclear import of nsP2 is necessary for suppressing the host antiviral response [134]. Interestingly, similar to that of DENV NS3, the nuclear localization of CHIKV nsP2 also occurs temporarily during early infection, after which this protein resides in the cytoplasm [136]. IFN antagonism by nsP2 can be achieved by several mechanisms, including via a reduction in the cGAS level by a global translational inhibition [131,136], an inhibition of STAT1 activation and/or block of pY-STAT1 nuclear import [133], and a promotion of STAT1 nuclear export [132].

### 3.2. Viruses That Replicate in the Nucleus

#### 3.2.1. Human Immunodeficiency Virus (HIV)

Human immunodeficiency virus (HIV), a member of the *Retroviridae* family, is an enveloped +ssRNA virus that was first recognized in 1981 as the causative agent of a new disease affecting T lymphocytes [137]. HIV-1 is more virulent and infectious compared to HIV-2; thus, HIV-1 is the leading cause of acquired immunodeficiency syndrome (AIDS) in the ongoing AIDS pandemic [138]. Upon viral entry, the viral replication complex undergoes reverse transcription followed by integration to form a pre-integration complex (PIC) before entering the nucleus (reviewed in [139]). Mutations in HIV proteins and the silencing of some NPCs can change the HIV integration pattern, which suggests that nuclear import is closely associated with the selection of a viral cDNA integration site for integration into the host genome (review in [140]).

The HIV-1 viral capsid was initially postulated to rapidly disassemble upon viral entry, releasing PICs into the cytosol, from which they subsequently translocate to the nucleus through NPCs [141]. A group of RNA interference (RNAi) screening studies have identified several Nups that are required for a HIV-1 infection, which include Nup98, Nup85, Nup133, Nup107, Nup160, Nup153, Nup214, Nup358, Nup155, Crm1, and the nuclear import receptor transportin 3 (TNPO3) [142,143,144]. In addition, a HIV-1 infection induces a downregulation in Nup50 and an upregulation in Nup62 for currently unknown reasons [145]. Some HIV-1 proteins, such as integrase (IN) [146], the viral protein R (Vpr) [147], and the matrix protein (MA) [148], contain NLSs. Notably, the width of the broad end of the HIV-1 capsid is approximately 60 nm [149], whereas the NPC pore size has long been thought to be only approximately 40 nm wide [19]. Collectively, these findings suggested that capsid disassembly is a prerequisite for PIC nuclear import.

More recent findings showing that the HIV-1 capsid remained assembled for at least the reverse transcription process [150] and that capsid uncoating can occur in the nucleus [151,152,153] have challenged the model described above. With a combination of different imaging tools, including cryo-electron tomography (cryo-ET) and subtomogram averaging, Zila and colleagues have visualized the entry of a whole HIV-1 capsid into a host nucleus through an NPC [154]. The diameter of an NPC measured in intact human cells (i.e., in a transporting state) was in fact larger than the diameter of an NPC measured in an isolated nuclear envelope (i.e., in a constricted state). The diameter of an NPC in the transporting state is larger than the broad end of the HIV-1 capsid; therefore, it can accommodate the import of a HIV-1 capsid into the nucleus. The team proposed a three-step process for the nuclear import of a HIV-1 capsid, during which the intact capsid interacts with different Nups in each stage. First, the HIV-1 capsids travel along microtubules to dock onto NPCs and interact with the FG-repeats and cyclophilin (Cyp) domain of Nup358 at the NPC cytoplasmic sites. Second, the intact capsids move deep into the central channels of the NPCs, which contain high local concentrations of FG-containing Nups within the Nup62 complex. Last, the capsids bind to Nup153 and cleavage and polyadenylation specificity factor subunit 6 (CPSF6). The HIV capsid–Nup153–CPSF6 interaction triggers capsid disassembly, which releases PICs into the nucleoplasm.

HIV Vpr suppresses the host antiviral response by interacting with α-importins (preferentially α5, but also, to a lesser extent, α1 and α4) to inhibit the IRF3 activation and to block the nuclear import of IRF3 and NfκB [155]. HIV Vpr has an N-terminal NLS and a C-terminal NLS [156]. Its C-terminal NLS was initially thought to be non-functional [156], but later studies have shown that it binds α-importins and mediates nuclear import without importin-β1 [157,158].

HIV-1 Rev has an arginine-rich NLS site in its N-terminal domain and a leucine-rich NES sequence in its C-terminal domain (reviewed in [159]). HIV-1 Rev enters the nucleus without binding RNA because only the RNA-free form of Rev can bind to importin-β1 for nuclear import [160]. The Rev–importin-β1 interaction is highly specific and can be blocked by importin-α5 [160]. The Rev NES is required for the nuclear export of unspliced viral RNA (vRNA). Rev binds to vRNA via the Rev response element (RRE) (reviewed in [161]). Rev contains an NES that allows it to bind to Crm1 and subsequently bring vRNA, in the form of a viral ribonucleoprotein (vRNP) transport complex, out into the cytoplasm in a RanGTP-dependent manner [162]. Several studies have suggested that Nup214, Nup153, Nup98, and Nup62 interact indirectly with vRNP through different Rev co-factors [163,164,165]. For example, the Rev–vRNP complex disturbs NPC integrity and causes a Nup62 cytoplasmic localization and subsequent encapsidation into a progeny virus [166]. A downregulation in Nup62 induced a vRNA nuclear accumulation, suggesting the importance of Nup62 in vRNA export [166]. The host Nxf1-dependent RNA export always involves active pre-mRNA splicing to prevent aberrant protein translation. Rev suppresses Nxf1-mediated vRNA export to secure a viral gene expression in the host [167]. McCauley et al. reported that an HIV-1 provirus used Crm1 and Rev to export its unspliced HIV for translation, which triggered chronic inflammation in patients with HIV who were receiving anti-retroviral therapy [168].

#### 3.2.2. Influenza A Virus (IAV)

Influenza A virus (IAV), a member of the *Orthomyxoviridae* family, is an enveloped, segmented −ssRNA virus that causes an epidemic respiratory disease (reviewed in [169]). IAV genomic RNAs are packed together with a viral nucleoprotein (NP) and a heterotrimeric RNA-dependent RNA polymerase (RdRp) complex (composed of PA, PB1, and PB2) into a rod-like vRNP (reviewed in [169]). Upon viral entry, free vRNPs translocate to the nucleus for viral RNA transcription and replication. The IAV NP and three components of the RdRp complex possess at least one NLS [170]. An IAV NP has two NLSs, a non-classical NLS in the N-terminus [171] and a classical bipartite NLS in the middle [172], and these are sufficient for vRNP nuclear import [170]. A mutation of the bipartite cNLS of the NP did not block its nuclear import, which suggests that the non-classical NLS of the NP is the predominant site [173] for its interaction with importins α5 and α7 [169]. Therefore, the non-classical NLS of an NP is essential for IAV replication [173]. Donchet et al. reported the binding affinity of an NP to different α-importins; an NP had the highest affinity for importin-α7 and the lowest affinity for importin-α1 [174].

IAV PB2 has a classical bipartite NLS [175], enabling its binding to the host α-importins (α1, α5, and α7) [176,177], preferentially importin-α7 [178]. Interestingly, both importins α1 and α7 act as positive regulators for PB2 polymerase activity, whereas importin-α3 acts as a negative regulator [177]. Highly pathogenic avian IAVs (HPAIVs) with human-like PB2 downregulate the expression of importin-α3, the main NTR for NF-κB, to suppress the host antiviral response [179]. The importin-β family member importin-5 (IPO5), also known as RanBP5, not only forms an import complex with PA and PB1 but also regulates the PA–PB1 complex for vRNA binding [180,181].

The Crm1-mediated nuclear export of newly synthesized IAV vRNPs is crucial for new viral synthesis. IAV vRNP, M1, and NES-bearing NS2 form a complex for vRNP export [182,183]. IAV NS1 exerts a negative regulatory effect on the host Nxf1-dependent mRNA export by downregulating Nup98 expression and forms an inhibitory complex with several of the host mRNA export factors (Nxf1, Nxt1, Rae1, and E1B-AP5) [184]. IAV NS1 binds at the FG-repeat binding site of Nxf1, where it interacts with the FG-domain of Nup98 during mRNA export [185]. In addition to disrupting the host mRNA export, IAV NS1 also interrupts the host mRNA processing, which it achieves by interacting with CPSF30 [186] and PABⅡ [187]. Collectively, these activities suppress the host protein biogenesis, particularly the factors required for an effective antiviral response [185]. Interestingly, in severe cases of influenza, a reduction in the protectin D1 (PD1) level enables IAV vRNA to bind directly to Nxf1, which then interacts with Nup62 for nuclear export. This mechanism does not require other mRNA export factors (e.g., Nxt1 or Crm1) or FG-containing Nups (e.g., Nup98 or Nup214) [188].

#### 3.2.3. Human Papilloma Virus (HPV)

Human papilloma virus (HPV), a member of the *Papillomaviridae* family, is a small, icosahedral, non-enveloped, double-stranded (ds)DNA virus [189]. In 1995, the International Agency for Research (IARC) classified HPV16 and HPV18 as human carcinogens because infection with these viruses increases the risk of developing cervical cancer [190]. HPV replicates in the host nucleus, and the entry of its viral genome into the host nucleus requires NE breakdown (during mitosis) rather than passage through an NPC [191,192]. 

The nucleocytoplasmic shuttling of E6 and E7, two oncoproteins found in high-risk HPVs, promotes carcinogenesis in HPV-infected cells. Only high-risk HPV E6 (E6_high_) can translocate to the nucleus [193] because its C-terminal contains three NLSs [193,194,195] that interact with importin-α1/β1, importin-β1, and importin-β2 [194]. Low-risk HPV E6 (E6_low_) predominantly resides in the cytoplasm, but E6_low_ acquires nuclear import activity when it is conjugated with an E6_high_ NLS [193]. The nuclear import of E6_high_ requires RanGDP, but GTP hydrolysis is not necessary [194]. Nuclear E6_high_ is critical for E6-mediated p53 degradation, independent of MDM2 [196]. E6_high_ mediates the polyubiquitination of p53, making it susceptible to proteosome degradation [197]. The E6_high_–p53 interaction promotes p53 export, and the p53 NES is required for a Crm1-mediated nuclear export [197]. The nuclear import of E6_high_ is compulsory for E6_high_-mediated p53 degradation; however, the p53 degradation can occur in both the nucleus and the cytoplasm [197].

To support viral DNA amplification, high-risk HPV E7 (E7_high_) must travel to the nucleus to hijack the host cell-cycle machinery, driving the host cell to re-enter the S-phase (reviewed in [198]). HPV16 E7 was initially thought to lack an NLS, and its nuclear import is Ran-dependent but independent of classical importin-α/β and importin-β2 receptors [199]. Knapp et al. later identified two NLSs in the *N*-terminal domain and CR3 domain of the HPV16 E7 protein [200]. Eberhard et al. found an explanation for the ability of the HPV16 E7 protein to undergo importin-independent nuclear import, despite having NLSs [201]. The zinc-binding domain within the E7 CR3 domain contains a hydrophobic patch (_65_LRLCV_69_) that enables E7 to accomplish its nuclear import by interacting with the FG-domain of Nup62 via a hydrophobic interaction [201]. Furthermore, this hydrophobic patch also facilitates the interaction of HPV16 E7 with Nup153 [202]. The hydrophobic interaction between HPV E7 and FG-containing Nups is conserved in other HPV serotypes (HPV8 and HPV11) [202,203]. A functional NES has been identified in HPV16 E7, which suggests that E7 nuclear egress is Crm1-dependent [200].

HPV encodes the E1 DNA helicase and the E2 original recognition protein and uses them to hijack the host DNA replication machinery for viral replication [204]. Therefore, the nuclear localization of E1 and E2 are important for efficient viral replication. The E1 proteins of several HPV serotypes (HPV11, HPV31, and HPV16) have bipartite NLSs [205], and these NLSs behave in a similar way to the bipartite NLS of the bovine HPV E1 protein [206], which can interact with multiple importins α (α1, α3, and α5) [207]. In addition, the phosphorylation of HPV E1 by the host ERK and/or JNK is needed for its nuclear import [205,206]. Regarding the HPV E1 nuclear export, this protein has a functional NES site, suggesting it undergoes a Crm1-dependent export [204,208]. This NES has a cyclin-dependent kinase (CDK) phosphorylation site, and NES phosphorylation by CDK causes HPV E1 to be retained in the nucleus, which is essential for viral replication [206,208].

The subcellular localizations of E2 proteins from low-risk (HPV11 and HPV6) and high-risk (HPV16 and HPV18) HPVs differ; low-risk HPV E2 is predominantly localized in the nucleus, whereas high-risk HPV E2 can be found in both the cytoplasm and the nucleus [209]. The NLS of the HPV11 E2 protein, but not those of the HPV16 and HPV18 E2 proteins, was found to have a dominant function [209]. Interestingly, cNLSs are found in high-risk HPV E2 but not in low-risk HPV E2; however, the NTRs for high-risk HPV E2 are still unknown [210]. The NES in high-risk HPV E2 enables the nucleocytoplasmic shuttling of this protein. Cytoplasmic accumulation of the high-risk HPV E2 proteins promotes caspase-8-mediated cell apoptosis [209].

The HPV L1 major capsid protein (L1) and L2 minor capsid protein (L2) are delivered to the nucleus for virion production. HPV16 L1 possesses both a monopartite and a bipartite NLS, and its nuclear import requires importin-α1/β1, RanGDP, and free GTP, but occurs independently of GTP hydrolysis [211]. The L1 protein also binds importin-β2, but RanGTP is unable to dissociate the complex [211]. This interaction inhibits the importin-β2-mediated nuclear import of hnRNP A1 [211]. An L2 protein nuclear import is required for HPV viral assembly [212]. For nuclear import, the HPV16 L2 protein can interact with importin-β2, importin-5, or the importin-α1/importin-β1 heterodimer. An NES has been identified in the L2 protein, but its function remains unclear.

#### 3.2.4. Hepatitis B Virus (HBV)

Hepatitis B virus (HBV), a member of the *Hepadnaviridae* family, is a small, enveloped dsDNA virus that propagates in hepatocytes [213]. Acute infection with HBV causes acute hepatitis, whereas chronic infection with HBV increases the risk of hepatocellular carcinoma (HCC) (reviewed in [214]). The HBV capsid disassembles in the nucleus, releasing relaxed circular DNA (rc)DNA, which is subsequently repaired by the host DNA repair machinery to form a covalently closed circular DNA, (ccc)DNA. The HBV cccDNA serves as a template for viral RNA replication (reviewed in [215]). Phosphorylation of the C-terminal HBV core or the capsid protein (Cp) exposes the Cp NLS, allowing the Cp to recruit classical importin-α/β1 receptors to mediate the nuclear import of the HBV capsid [216,217]. In addition to phosphorylation, the completion of the (+) DNA and removal of viral DNA polymerase from rcDNA triggers a conformational change of the Cp, exposing the C-terminal NLS site [218]. During its translocation through an NPC, RanGTP triggers the dissociation of the Cp-importin-α/β1 ternary complex, which allows for an interaction between the Cp and Nup153 [219]. Notably, Nup153 is the sole FG-containing Nup that interacts with the Cp, which suggests that this interaction is not mediated by a hydrophobic interaction [219]. Finally, mature HBV capsids disintegrate in a Ran-independent manner [217], releasing viral rcDNA; capsid disassembly is halted in immature capsids [219].

The Cp has the following four arginine-rich domains (ARDs): ARD Ⅰ and Ⅲ each behave in a similar way to an NLS, and ARD Ⅱ and Ⅳ each behave in a similar way to an NES [220]. The Cp NES is needed for an Nxf1-dependent viral pre-genomic RNA (pgRNA) export [220]. The nuclear export of pgRNA is a critical step for new virion synthesis. The subcellular localization of the Cp is rather complex, depending on both the intrinsic factors (NLS and NES) and the extrinsic factors (importins, Nxf1, and cellular kinase) [220]. A recent study showed that increasing the nuclear Cp concentration induces Cp export in a Crm1-dependent manner, which suggests that the Cp concentration may manipulate different export signals [221]. An empty Cp can interact with importin-β1 independent of importin-α via its IBB domain, but the exact biological function of this interaction is yet to be elucidated [222]. Mitra and colleagues reported that the cytosolic HBV e antigen (HBeAg), also known as the precore protein intermediate (p22), interacts with importin-α5 through its C-terminal ARD. This interaction blocks the pY-STAT1 nuclear import and subsequently suppresses the host IFN response [223].

#### 3.2.5. Herpes Simplex Virus Type-1, Human Cytomegalovirus, and Epstein–Barr Virus

Herpes simplex virus type-1 (HSV-1) is a dsDNA α-herpesvirus that causes cold sores in human beings [224]. After internalization, the viral capsid travels along microtubules to reach the nucleus to release a viral genome into the nucleoplasm for transcription and translation [225]. The tegument proteins VP1/2 [226] and pUL25 [225] interact with Nup358 and Nup214 to dock the viral capsid on an NPC. VP1/2 has an efficient NLS at its N-terminal, but the protein is mainly located in the cytoplasm [227]. The NLS of VP1/2 is specific to importin-β1 rather than importins α [227,228], and VP1/2-importin-β1 interaction is important for directing the viral capsid to dock on an NPC [227]. Using mouse embryonic fibroblast (MEF) cells, Döhner et al. comprehensively demonstrated the distinct importin-α isoforms (α1, α3 and α4) in mediating HSV-1 gene expression, nuclear localization of viral proteins, capsid assembly, and capsid egress [229]. Importins are not needed for the NPC docking of an HSV-1 capsid, for the nuclear import of incoming HSV-1 genomes, or for the nuclear translocation of the HSV-1 VP16 protein. Importins α indirectly regulate the HSV-1 protein expression, and within this regulation importin-α1 is facilitative but importin-α4 is restrictive. Importin-α1 and Importin-α3 orchestrate the nuclear localization of immediate-early (ICP4 and ICP0) and early (ICP8 and pUL42) HSV-1 proteins. The HSV-1 DNA polymerase subunits pUL30 and pUL42 utilize several nuclear import mechanisms. HSV-1 pUL30 has a non-canonical and a classical bipartite NLS, and interacts with importin-α5, but interactions with other importin-α isoforms remain unknown [230,231,232]. HSV-1 pUL42 has a bipartite NLS, and the NLS binds mainly to importin-α7 and, to a lesser extent, to importin-α1, but not to importin-α3 [233]. Interestingly, the mutation of the NLS in either pUL30 or pUL42 does not impede the nuclear import of both proteins under the holoenzyme form [233]. The holoenzyme only resides in cytosol if the NLSs in both pUL30 and pUL42 are mutated [233]. Importin-α1 is crucial for an HSV-1 infection because importin-α1 is required for efficient capsid assembly and egress to make new virions in cytosol. The team also illustrated that silencing either importin-α1 or importin-α3 is sufficient to suppress HSV-1 gene expression in terminally differentiated cells, neurons for example, but HSV-1 gene expression remains unperturbed in MEF cells [229]. MEF is not a terminally differentiated cell type [234]. Such a discrepancy indicates that importins α repertoire in MEF is sufficient to compensate for the absence of importin-α1 or importin-α3 to sustain HSV-1 gene expression.

HSV-1 ICP27 is an immediate-early protein that is required for enhancing HSV-1 gene expression and for exporting intronless HSV-1 mRNAs (review in [235,236]). Different conformations of ICP27 confer different specificities/preferences for nuclear export. Viral mRNA-bound ICP27 interacts with Aly/REF to recruit Nxf1 to export intronless HSV-1 mRNA for translation [237]. Nonetheless, Aly/REF knockdown does not significantly dampen viral mRNA export [238], suggesting that ICP27 could utilize other host export factors to compensate for Aly/REF. Free ICP27 does not require both Nxf1 and Crm1 for its export [239]. Instead, its N and C termini interact with Nup62 to enable ICP27 shuttles between the cytoplasm and the nucleus. The nuclear import of ICP27 inhibits both classical, importin-α/β1-dependent, and transportin-dependent nuclear import [240]. Beside the main export receptor (Nxf1), a recent finding reveals that the HSV-1 integral protein, glycoprotein M (gM), binds to exportin-6 (XPO6) for nuclear export to the trans-Golgi network (TGN) [241].

Human cytomegalovirus (HCMV) is a ds-DNA β-herpesvirus that is associated with mononucleosis syndrome (review in [242]), venous thromboembolism (VTE) (review in [242]), and cytomegalovirus encephalitis in immunocompromised patients (review in [243]). HCMV pUL97 is a multifunctional protein kinase that phosphorylates CDK1 and modifies the host G2/M cell cycle checkpoint regulators to promote viral replication [244]. There are two isoforms of pUL97, large and small isoforms. The large isoform has two bipartite NLSs (NLS1 and NLS2), whereas the small isoform only has NLS2, located at their N-terminals [245]. Therefore, the large isoform has a higher nuclear localization efficiency compared to the small isoform [245]. Only importin-α1 has been shown to interact with pUL97, and other importin-α isoforms have not been tested [245]. HCMV pUL79 is an elongation factor of RNA polymerase II for viral gene transcription during the late stages of HCMV infection [246]. HCMV pUL79 possesses a hydrophobic PY-NLS, which enables it to translocate to the nucleus through an importin-β2-mediated pathway [247]. The multifunctional protein, HMCV pUL84, is believed to initiate lytic viral DNA synthesis [248]. Lischka et al. performed in vitro transport assays and found that the nuclear import of pUL84 relies on the classical importin-mediated import pathway [249]. Although pUL84 has a putative NLS, the NLS does not have the NLS activity [249]. Instead, a large domain with 282 amino acids is needed for an importin-α–pUL84 interaction [249]. A team has shown that pUL84 interacted with several importin-α isoforms, including α1, α 3, α 4, and α5 [249]. Interestingly, the pUL84–importin-α interaction domain also contains two leucin-rich NESs, and this region allows the nuclear export of pUL84 via the Crm1-dependent pathway [250]. Gao and his coworkers conducted RNA pulldown assays to reveal pUL84′s viral mRNA export activity [248]. The team reported that pUL84 interacted with a viral-encoded transcript known as IRS1 and mediated the cytoplasmic localization of an IRS1 transcript [248].

Similar to HSV-1 ICP27, HCMV encodes the pUL69 regulatory protein to mediate a viral mRNA export. The arginine rich RNA-binding domain and the DExD/H-box helicase UAP56-binding motif in pUL69 [251] allows pUL69 to interact with the mRNA export factor UAP56/URH49 [252], transcription elongation factor hSPT6, and intronless viral mRNA to form a messenger ribonucleoprotein (mRNP) for an Nxf1-Nxt1-dependent mRNA export (reviewed in [253]). An NES is required for the nuclear export of free pUL69 but independent of Crm1, and the exact export mechanism remains elusive [254]. Likewise, in silico analysis failed to determine a classical NLS within pUL69, the NTRs associated with pUL69 nuclear import are also unknown [252]. CDK9 phosphorylation of pUL69 is crucial for a pUL69-mediated viral mRNA export because CDK inhibition triggers the nuclear accumulation of pUL69 and suppresses the mRNA export activity of pUL69 [255].

The *Herpesviridae* family member Epstein–Barr virus (EBV), also known as human herpesvirus 4, is a dsDNA γ-herpesvirus that is associated with the development of three types of B-cell lymphoma (Burkitt’s lymphoma, Hodgkin’s lymphoma, and diffuse large B-cell lymphoma) and of nasopharyngeal carcinoma (NPC) (reviewed in [256]). Viral replication, which occurs in the host nucleus, has two different replication patterns, latent replication (during B-cell proliferation) and lytic replication (virion production) (reviewed in [256]). In latent replication, EBV nuclear antigen 1 (EBNA1) is the only viral protein needed in the host nucleus for viral DNA retention [257]. EBNA1 has an NLS (379KRPRSPSS386) that binds to importin-α1 and importin-α5 for its nuclear import [257]. In lytic replication, several viral proteins, including the DNA-replicating enzymes BSLF1, BBLF2/3, BBLF4, and a major capsid protein (VCA), are translocated to the nucleus for viral DNA replication and nucleocapsid assembly [258]. However, these viral proteins do not possess canonical NLSs. To circumvent this limitation, EBV expresses the BGLF4 protein kinase to facilitate nuclear translocation, probably by inducing the nuclear accumulation of RanGAP1 to inhibit the nuclear import of cNLS-bearing cargoes, phosphorylating FG-containing Nups (Nup62 and Nup153) to dilate the NPC, and inducing microtubule reorganization to change the nuclear shape [258]. The BGLF4 homolog proteins Herpes Simplex 1 (HSV-1) UL13 (α-herpesvirus), human Cytomegalovirus (HCMV) UL97 (β-herpesvirus), Kaposi’s sarcoma-associated herpesvirus (KSHV) ORF36 (γ-herpesvirus), and Murine Gammaherpesvirus 68 (MHV68) ORF36 (γ-herpesvirus) promote nuclear lamina disassembly via a BGL4-like mechanism [259,260]. Intriguingly, only γ-herpesvirus BGL4 homolog proteins (KSHV ORF36 and MHV68 ORF36) can mediate the nuclear import of VCA [258].

The nuclear translocation of EBV EGR1 is mediated by importin-7 (IPO7) [261], and the accumulation of EGR1 is correlated with the viral lytic phase [262]. EGR1 can negatively regulate IPO7 expression using EBV miRNAs (mirBART3 and mirBART16) to maintain a level that is optimal for the growth of EBV-transformed cells [261]. EBV EB2 (also called M or SM) interacts with Crm1 to boost the EB2-mediated gene expression and also to mediate the export of unspliced lytic EBV mRNA [229]. EB2 was shown to be associated with the GTPase Ran and Nup214 during the export of EBV mRNA to the cytoplasm [263]. Herpesviridae family members express nuclear egress proteins 1 (BFLF1) and 2 (BFRF2) to form a nuclear egress complex (NEC) at the host inner NE, which allows the export of new viral capsids containing viral DNA [264,265,266]. EBV BFLF2 interacts with EBV BFRF1 and lamin B at the nuclear rim, making a nuclear lamina for new EBV maturation and budding through the inner nuclear membrane [265]. The homolog proteins of BFLF2, HSV-1 UL31 (α-herpesvirus) [264] and HCMV UL53 (β-herpesvirus) [266], also possess NLSs to mediate their nuclear import. HSV-1 UL31 has a functional bipartite NLS [264], whereas both HCMV UL53 [266] and EBV BFLF2 [267] have a functional monopartite NLS. BFLF2’s nuclear import requires Ran, importin-α7, importin-β1, and importin-β2, but not importins α1, α3, or α5 [267]. On the other hand, HSV-1 UL31 interacts with Ran, importin-α1, and importin-β2 for its nuclear import [268,269].

Non-functional NESs have been found in EBV BFLF2 [267] and HSV-1 UL31 [264,269], and no NESs have been found in HCMV UL53 [270]; these findings suggest that these proteins do not require Crm1 for their export. EBV BFLF2 interacts with Nxf1 in the absence of RNA for its export. The nuclear export receptors for HSV-1 UL31 and HCMV UL53 are yet to be determined [267]. Funk et al. conducted a comprehensive analysis on the EBV and HSV-1 tegument proteins using an in vitro assay called Nuclear EXport Trapped by RAPamycin (NEX-TRAP) [271] and found that two EBV (pBTRF1 and pBGFL3) and nine HSV-1 tegument proteins showed nuclear export activity. The group compared the nuclear export activity between the EBV and HSV-1 tegument orthologs; EBV pBTRF1 behaved in a similar way to its HSV-1 orthologue pUL21, exhibiting active export activity. Conversely, EBV pBGFL3 (exported) displayed the opposite behavior compared to its HSV-1 orthologue pUL14 (non-exported). The NES of pBTRF1 matches the Rev NES consensus, and that of pBGFL3 matches the PKI NES consensus. The NESs of both Rev and PKI are recognized by Crm1, which suggests that EBV pBTRF1’s and pBGFL3’s export activities are Crm1-dependent. A leptomycin B (LMB) assay revealed six HSV-1 tegument proteins (pUL4, pUL11, pUL13, pUL21, pUL37d11, and pUL48) that are exported in a Crm1-dependent manner.

## 4. Potential Antiviral Drugs That Target the Host Nuclear Transport Machinery

The exploitation by viruses of the host nucleocytoplasmic trafficking is crucial for viral genome replication, viral component assembly, suppression of the host antiviral response, and cellular state alteration. To date, viral polymerase inhibitors are actively used in clinics against various RNA and DNA viruses including EBOV [273], HIV [274], and HPV [275]. In addition to viral polymerase inhibitors, nuclear transport inhibitors can also be considered to reduce the viral load and/or ameliorate clinical symptoms, either in monotherapy or in combination with other antiviral agents. In this section, we discuss the host-specific and the viral-specific nuclear transport inhibitors and relevant clinical trials. The key points are summarized in Table 3 and Table 4.

### 4.1. Host-Specific Nuclear Import Inhibitors

The importin-α/β1 heterodimer is a common NTR used by many viruses for nuclear entry. Importin-α inhibitors include Bimax (1 and 2) [276], cSN50.1 [277,278], Ivermectin [279,280], and GW5074 [281]. Among these drugs, only cSN50.1 is an importin-α isoform-specific inhibitor; it targets importin-α5 [278]. Both of the small molecule inhibitors, Ivermectin [282] and GW5074 (a c-Raf inhibitor) [281], share the same inhibitory mechanism. They bind to importin-α to block cargo loading and the formation of the importin-α/β1 heterodimer. Ivermectin has a broad-spectrum in vitro and/or in vivo antiviral effect against different types of RNA and DNA viruses, including SARS-CoV-2 (see Table 3). GW5074 has in vitro antiviral activity against two types of flaviviruses, DENV2 and ZIKV [282].

Importin-β inhibitors can directly block the formation of the cargo-receptor ternary complex. Several importin-β inhibitors, including three importin-β1 inhibitors (Importazole [283], INI-43 [284], and Karyostatin [285]) and the importin-β2 inhibitor M9M [286], have been studied for their nuclear import blockade activity in cancer cells. Thus far, only M9M has been tested for its antiviral effects; it was found to block the nuclear import of HSV-1 UL6 [287].

### 4.2. Host-Specific Nuclear Export Inhibitors

As Crm1 is crucial for the nuclear export of a vast number of cargo types, it is a main target of viruses for the export of their proteins. LMB and its derivatives [288,289], as well as selective inhibitor for nuclear export (SINE) [290,291] compounds, target a common site of Crm1, Cysteine-528, to block Crm1 from interacting with the NES-bearing cargo. After it binds to Crm1, LMB undergoes hydrolysis and subsequently forms a salt bridge in LMB–Crm1, irreversibly inhibiting Crm1’s function [292]. Conversely, SINEs do not undergo hydrolysis after they bind to Crm1; therefore, there is no salt bridge formation [293,294]. However, the SINE–Crm1 interaction promotes Crm1 degradation, and Crm1 re-synthesis occurs after SINE administration is discontinued [295]. Clinical trials of LMB were halted because the treatment caused profound adverse effects, presumably owing to the irreversible LMB–Crm1 interaction [296]. Conversely, SINEs have shown much lower toxicity in clinical trials because their inhibition of Crm1’s function is reversible (reviewed in [297]). The SINEs Selinexor and Verdinexor, which have been actively tested in cancer patients, are now under evaluation for their antiviral activity in in vitro and in vivo settings (Table 3).

### 4.3. Viral-Specific Nuclear Transport Inhibitors

*N*-(4-hydroxyphenyl) retinamide (4-HRP or Fenretinide) targets Flavivirus polymerase NS5 and prevents NS5 nuclear import. 4-HRP has both therapeutic and prophylactic potential against DENV and ZIKV [298,299,300]. Not only is 4-HRP effective against four DENV serotypes, but it also prevents the antibody-dependent enhancement (ADE) of dengue hemorrhagic fever (DHF) [300]. A blockade of ZIKV NS5 nuclear accumulation in neurons could protect neurons from inflammation, thus reducing the risk of neurological disorders due to ZIKV infection [123].

Two potent HIV capsid inhibitors, GS-6207 and GS-CA, have been developed by Gilead Science [301,302]. These small molecule inhibitors bind to the HIV capsid protein, impeding the delivery of viral PIC into the host nucleus and thus interrupting capsid assembly and viral particle production. The binding site of the inhibitors is the site where Nup153 and CPSF6 bind during the HIV capsid’s nuclear entry.

Mohl et al. developed a model for an IAV PB1/PA–RanBP5 complexation, and then used PPI software-based virtual screening to identify potential inhibitors that target IAV PB1 and block its interaction with importin-5 (RanBP5) [303]. In vitro studies have shown that five compounds may inhibit the nuclear localization and in vitro polymerase activity of PB1/PA.

Some groups have applied in silico screening to identify inhibitors of EBOV VP24 and importin-α isoforms. Tanaka et al. used a CE-SELEX system to select oligonucleotide-based VP24-binding aptamers that inhibit the VP24–importin-α1 interaction [304]. Two of these inhibitors, VPKS-2 and VPKS-5, were found to inhibit the VP25–importin-α5 interaction in vitro. Another research team, Song et al., utilized the Random non-standard Peptides Integrated Discovery (RaPID) system to design high-affinity macrocyclic peptides that compete with importin-α6 for VP24 binding [305]. These strategies protect importins α from VP24, and thus restores the host antiviral defense system during infection.

The therapeutic effects of interferon β (type-I interferon) on recurrent HPV infection lesions have been clinically proven, especially in patients with cervical intraepithelial neoplasia (CIN) [306]. Type Ⅰ IFN strongly induces p56 expression, and p56 binds the E1 protein of several HPV serotypes [307]. In vivo experiments demonstrated that cytoplasmic p56 promotes the nuclear export of the E1 protein [307]. Other in vitro experiments revealed that recombinant p56 inhibits the DNA helicase activity of E1, consequently halting HPV DNA replication [307].

The nucleocytoplasmic trafficking of HBV core/capsid proteins is required for HBV to successfully infect adjacent healthy hepatocytes and to persistently re-infect HBV-infected hepatocytes. Capsid assembly modulators (CAMs), including heteroaryldihydropyrimidines (HAPs) [308,309,310], phenylpropenamides (PPAs) [311,312,313], and sulfamoylbenzamides (SBAs) [314], are studied for their anti-HBV efficacies. CAMs can reduce HBV cccDNA levels through several mechanisms. For example, they can block the nuclear entry of HBV nucleocapsids, which circumvent persistent infection in HBV-infected cells or inhibit cccDNA formation in newly HBV-infected cells [315,316], and they can disrupt the structural integrity of cccDNA (reviewed in [317]).

### 4.4. Clinical Translation of Nuclear Transport Inhibitors against Viral Infections

Regarding the importin-α inhibitors, only Ivermectin has been assessed in clinical studies for its antiviral efficacy. Three clinical studies have been performed to evaluate the clinical benefits of Ivermectin in patients with COVID-19 [325,326,327]. Two of these clinical studies (one randomized clinical trial (RCT) and one non-RCT) found that Ivermectin, either in monotherapy [327] or in combination therapy [326], significantly improved the viral clearance in patients with mild cases of COVID-19. However, another clinical study on Ivermectin monotherapy found that it did not shorten the time required for symptom resolution in patients with mild cases of COVID-19 [325]. The observed clinical and immunological spectra of patients with COVID-19 indicate that viral clearance may not directly correlate with symptom resolution because symptoms that are induced by a dysregulated immune response can persist after successful viral clearance [328]. The synergism between Ivermectin and Remdesivir for treating COVID-19 has not yet been evaluated. In patients with dengue fever, a phase two RCT trial showed that Ivermectin monotherapy promoted NS1 antigenemia clearance but not viral clearance [329]. The serum NS1 level is positively correlated with dengue hemorrhagic fever (DHF) [330]. The role of Ivermectin in reducing the risk of DHF development in patients with dengue fever has not yet been investigated.

Two SINE candidates, Selinexor and Verdinexor, are actively being tested as therapeutics in cancer patients. Currently, one clinical trial (NCT04349098) has completed to determine the clinical benefits of Selinexor in treating moderate-to-severe cases of COVID-19. Additionally, a recently completed phase one clinical trial (NCT02431364) reported that Verdinexor exhibited an improved adverse effect profile in healthy volunteers. However, the study has been terminated for administrative reasons.

Viral factor-specific nuclear transport inhibitors, such as GS-6207, REBACIN^®^, and CAM (NVR 3-778 and GLS4), are currently being tested in clinical trials. A phase one clinical trial of GS-6207 (NCT07379866) found that a single dose of subcutaneously administered GS-6207 effectively reduced the plasma HIV viral load and that the protection could persist for more than six months [301]. In two parallel RCTs, REBACIN^®^ was found to induce a strong viral clearance in HPV-positive patients [331]. Mechanistically, REBACIN^®^ inhibits the mRNA transcription of high-risk HPV E6 and E7 oncoproteins in mice [331]. In addition, Yang et al. performed a retrospective analysis and a meta-analysis and found that REBACIN^®^ showed better efficacy than IFN for treating a persistent high-risk HVP infection [332]. Yuen et al. performed a phase one RCT to test the anti-HBV activity of an SBA class CAM, NVR 3-778, in patients with chronic HBV infection without cirrhosis [333]. It revealed that NVR 3-778 was well-tolerated by patients and that this CAM compound could reduce the viral load (as measured by viral DNA and RNA levels); furthermore, its administration with pegylated IFN (pegIFN) produced a synergetic antiviral effect [333]. Zhang et al. reported that a HAP class CAM, GLS4, demonstrated antiviral activity in patients with a chronic HBV infection in a phase 1b study, and that this drug was well-tolerated by patients [334].

## 5. Conclusions

Viral proteins masquerade as host factors to exploit the host nuclear transport machinery for viral replication, and they reprogram the host cellular environment to enhance their replication and evade the host immunity. Nuclear transport inhibitors can not only interrupt viral replication but also disrupt the viral assembly and restore the host immunity. Targeting host factors to block nuclear transport could exert a broad-spectrum antiviral effect, as reported for Ivermectin. Nonetheless, the feasibility of using inhibitors of host factors as antiviral drugs could be limited when they target important NTRs, such as importin-β1 and Crm1, owing to likely adverse effects. Furthermore, many infectious diseases, especially COVID-19, have a complicated clinical spectrum. The administration timing of the host-specific nuclear transport inhibitors needs to be carefully evaluated to maximize clinical benefits. In silico drug screening and in vitro and in vivo experiments could accelerate the identification of potent viral-specific nuclear transport inhibitors. As certain viral factors not only exploit the host NTRs but also have their own biological functions, viral-specific nuclear transport inhibitors could “kill two birds with one stone”, that is, not only improve the viral clearance but also preserve/restore the host nucleocytoplasmic trafficking. Lastly, on the basis of our experience applying HS-AFM imaging to human NPCs [17,336], DNA–DNA-binding protein interactions [337], and viral proteins [338,339], we believe it will be worthwhile to observe the interaction between the viral factors and the host nuclear transport factors in real-time.

## Figures and Tables

**Table 1:** List of the nuclear transport receptors (NTRs) involved in nucleocytoplasmic shuttling.

Adaptor Protein Importin α Subfamilies	Protein Name (Importin Nomenclature)	Protein Name (Karyopherin Nomenclature)
α1	Importin α1 Importin α8	Karyopherin α2 Karyopherin α7
α2	Importin α3 Importin α4	Karyopherin α4 Karyopherin α3
α3	Importin α5 Importin α6 Importin α7	Karyopherin α1 Karyopherin α5 Karyopherin α6
**Importin β family members**	**Cargoes**	
Importin β1 (Karyopherin β1)	Importin α isoforms-cNLS-bearing cargoes Snurportin 1 RIPα (RPA-interacting protein α) Importin 7	
Importin β2 (Transportin-1)	hnRNP (A1, A2, and F) Ribosomal proteins TAP/Nxf1 Histones	
Transportin-2	HuR (ELAV-like protein 1)	
Transportin-SR	SR proteins (abundant arginine/serine-rich proteins)	
Importin 4 (IPO4, RanBP4)	Histones and ribosomal proteins	
Importin 5 (IPO5, RanBP5)	Histones and ribosomal proteins	
Importin 7 (IPO7, RanBP7)	HIV PIC H1 histone GR (Glucocorticoid receptor) Ribosomal proteins	
Importin 8 (IPO8, RanBP8)	SRP19 (Signal recognition particle 19)	
Importin 9 (IPO9, RanBP9)	Histones and ribosomal proteins	
Importin 11 (IPO11, RanBP11)	UBE2E3 (Ub-conjugating enzyme) rpL12 (60S ribosomal protein L12)	
Crm1 (XPO1)	Cargoes with leucine-rich NES Snurportin HuR (ELAV-like protein 1) [62] Nxf3 (Nuclear RNA export factor 3) [63] LRPPRC (Leucine-rich PPR motif-containing protein) [64] Nmd3 (60S ribosomal export protein) [65]	
CAS (XPO2)	Importin α	
Exportin 4 (XPO4)	eIF-5A (Eukaryotic translation initiation factor 5A-1)	
Exportin 5 (XPO5)	ILF3 (Interleukin enhancer-binding factor 3) eEF1A-1 (Elongation factor 1-alpha 1) pre-miRNA, tRNA, minihelix RNA	
Exportin 6 (XPO6)	Profilin Actin	
Exportin-T (XPOT)	tRNA	
Importin 13 (IPO13, RanBP13), import	RBM8A (RNA-binding protein 8A) UBCE9 (SUMO-conjugating enzyme UBC9) Pax6 (Paired box protein Pax-6)	
Importin 13 (IPO13, RanBP13), export	eIF-4C (Eukaryotic translation initiation factor 1A, X-chromosomal)	
RanBP6	Unknown	
RanBP16	Unknown	
RanBP17	Unknown	

Importins α: adapted from a review by Pumroy et al. [20]. Importins β: adapted from a review by Mosammaparast et al. [21] and other studies [64,65,66,67]. Yellow indicates nuclear import, orange indicates nuclear export, green indicates bidirectional shuttling, and blue indicates unknown cargo. Only human cargoes are mentioned here.

**Table 2:** Interactions between viral factors and host factors in the hijacking of the host nuclear transport machinery.

Viral Family	Site of Replication	Virus Name	Viral Factors	Host Factors	Effect of Viral–Host Factor Interaction	References
*Coronaviridae* (+ssRNA)	Cytoplasm	SARS CoV-2	Orf6	Importin-α1/β1	Blocks nuclear translocation of IRF3 to suppress IFNβ production.	[75,76,78]
				Importin-α/β1 (isoform α1, α5)	Blocks nuclear translocation of activated STAT1/2 to suppress IFN-mediated antiviral response.	
				Nup98 and Rae1	Disrupts importin-α5/β1 docking on Nup98-Rae1 complex for nuclear import.	
					Cytoplasmic accumulation of Nup98 and Rae1.	
					Nuclear accumulation of hnRNP A1.	
			Nsp1	Nxf1	Interrupts Nxf1 binds to mRNA export adaptors including Aly/REF and UAP56.	[83]
					Reduces interaction between Nxf1 and Nups (Nup98, Nup214, Nup358).	
		SARS CoV-1	Orf6	Importin-α1/β1	Connects importin-α1/β1 at ER/Golgi to sequester the receptor for activated STAT1 nuclear import.	[79,80]
			Nsp1	Nup93	Mislocalization of Nup93 (from NE to cytoplasm).	[84]
				Nucleolin	Cytoplasmic accumulation with unknown reasons.	
			Orf9b	Crm1	Nuclear export of Orf9b prevents Caspase-3-mediated apoptosis.	[85]
		MERS CoV	Orf4b	Importin-α3/β1	Blocks IFR3 and IRF7 nuclear import for IFNβ production.	[86,87]
					Blocks NF-κB p65 subunit nuclear import to suppress host antiviral response.	
*Filoviridae* (-ssRNA)	Cytoplasm	Zaire EBOV	VP24	Importin-α/β1 (isoform: α5, α6, and α7)	Blocks nuclear translocation of activated STAT1 to suppress IFN-mediated antiviral response.	[91,97]
				Importin-α5/β1	Cytoplasmic accumulation of hnRNP C1/C2 for viral replication.	
			Inclusion body (IB)	Importin-α7	Required for IB formation.	[104]
			Nucleo-protein (NP)	Nxf1	Exports viral mRNA from IB to cytoplasm for translation	[105]
*Flaviviridae* (+ssRNA)	Cytoplasm	DENV	NS5	Importin-α2/β1 (DENV serotype 2 and 3 only)	Nuclear import of NS5 has unclear functions on viral replication and pathogenesis of the disease.	[115,116]
				Crm1	Nuclear export of NS5 promote IL-8 production and suppress viral replication.	
			NS3	FG-Nups (Nup62, Nup153, and Nup98)	Works in concert with NS2B3 to degrade FG-Nups.	[117]
		ZIKV	NS5	Importin-α/β1 (isoform α1, α 3, α4, and α 7)	Protects NS5 from cytoplasmic degradation.	[122]
					Sequesters importins α in nuclear bodies.	[123]
			NS3	Nups (TPR, Nup153, and Nup98)	Works in concert with NS2B3 to degrade Nups.	[117]
*Togaviridae* (+ssRNA)	Cytoplasm	CHIKV	Capsid protein (CP)	Importin-α3/β1	Purpose of nuclear localization of CP is unclear.	[129,130]
				Crm1	Mutation in CP NES blocks host nuclear import with unknown mechanisms.	
			nsP2	nil	nsP2 does not have NLS but it can enter nucleus to suppress host immunity.	[131,135]
*Retroviridae* (+ssRNA)	Nucleus	HIV	Viral Capsid (CA)	Nup358 and Nup62	Nuclear import of viral capsid.	[154]
				Nup153 and CPSF6	Disassembles viral capsid to release PIC to nucleus.	
			Vpr	Importin-α/β1 (preferably to α5, lesser extend to α1 and α4)	Inhibits IRF3 activation and to block nuclear import of IRF3 and NfκB.	[155]
			Rev	Importin β1	Rev nuclear import is needed to form viral ribonucleoprotein (vRNP) transport complex, and the complex is then exported out by Crm1.	[160,162,163,164,165]
				Crm1		
				Nups (214, Nup153, Nup98, and Nup62)		
*Orthomyxoviridae* (-ssRNA)	Nucleus	Influenza A	Nucleo-protein (NP)	Importin-α/β1 (isoform: α5, α7)	vRNP nuclear import for viral replication.	[170,173]
			PA-PB1 (RdRP subunit)	Importin 5	Forms an import complex with PA and PB1, but also regulates PA-PB1 complex for vRNA binding.	[180,181]
			PB2 (RdRP subunit)	Importin-α/β1 (isoform: α1, α5, α7)	vRNP nuclear import for viral replication.	[176,177,178]
					Importin-α isoform switching for viral adaptation in different host species.	[272]
				Importin-α3/β1	Negatively regulate PB2 polymerase activity.	[177]
					Downregulates importin-α3 expression to suppress host antiviral response by blocking NF-κB nuclear import.	[179]
			NS2	Crm1	Forms an export complex with vRNP, viral M1 protein.	[182,183]
			NS1	Nup98	Downregulation of Nup98 expression to inhibit host mRNA export.	[184]
				Nxf1	Binds to FG-repeat binding site of Nxf1 to block host mRNA export.	
			vRNA	Nxf1	In severe influenza infection, vRNA binds to Nxf1 alone and interacts only with Nup62 for nuclear export.	[188]
				Nup62		
*Papillomaviridae* (dsDNA)	Nucleus	HPV	High-risk E6	Importin-α1/β1, Importin- β1, Importin-β2	Mediates polyubiquitination of p53 for proteasome degradation independent of MDM2.	[194,196,197]
			High-risk E7	FG-Nups (Nup62 and Nup153)	Re-programs cell cycle to S-phase to support viral DNA amplification.	[202]
				Crm1	Nuclear export of E7.	[200]
			E1	Importin-α/β1 (isoform: α1, α3, α5)	Phosphorylation of E1 by host ERK and/or JNK is needed to enable E1 binds importins α for nuclear import for viral replication.	[205,206,207]
				CDK	Phosphorylation of E1 blocks Crm1-dependent export to retain E1 in nucleus for viral replication.	[204,208]
			E2	Unknown import NTR	Nuclear import of E2 is needed for viral replication.	[210]
				Crm1	Cytoplasmic accumulation of high-risk E2 induces caspase-8 mediated cell apoptosis.	[209]
			L1 major capsid	Importin α1/β1	Capsid proteins assembly in nucleus.	[211]
				Importin β2	Inhibits nuclear import of hnRNP A1.	
			L2 minor capsid	Importin α1/β1	Capsid proteins assembly in nucleus.	[212]
				Importin β2		
				Importin 5		
*Hepadnaviridae* (dsDNA)	Nucleus	HBV	Capsid protein (Cp)	Importin α1/β1	Viral capsid binds to classical importin α/β1 to pass through NPC. Capsid disassembly starts once it interacts with Nup153.	[216,217,219]
				Nup153		
				Nxf1	pgRNA export for virion production.	[220,221]
				Crm-1	pgRNA export for virion production (when nuclear Cp concentration is high).	
			p22 (HBeAg)	Importin-α5/β1	Blocks activated STAT1 nuclear import to suppress host antiviral response.	[223]
*Herpesviridae* (dsDNA)	Nucleus	HSV-1	VP1/2	Importin-β1	Viral capsid docking on NPC to deliver viral DNA to nucleus.	[225,226]
			pUL25	Nup358, Nup214		
			pUL30	Importin-α5/β1	Nuclear localization of pUL30 and pUL42 is needed for viral replication in host nucleus.	[230,231,232,233]
			pUL42	Importin-α/β1 (isoform: α1 and α7)		
			ICP27	Aly/REF, Nxf1	Viral mRNA export for viral protein translation.	[237]
			gM	Exportin-6 (XPO6)	Nuclear export of gM to TGN.	[241]
		HCMV	pUL97	Importin-α1/β1	Phosphorylates CDK1 and modifies G2/M cell cycle checkpoint regulators to promote viral replication.	[244,245]
			pUL79	Importin-β2	Function as an elongation factor of RNA polymerase II for viral gene transcription in nucleus.	[246,247]
			pUL84	Importin-α/β1 (isoform α1, α3, α4, and α5)	Initiate lytic viral DNA synthesis in nucleus.	[248,249]
				Crm1	Viral mRNA export (IRS1 transcript)	[248]
			pUL69	UAP56/URH49, hSPT6, Nxf1	Viral mRNA export	[253]
		EBV	EBNA1	Importin-α/β1 (isoform: α1, α5)	Nuclear EBNA1 tethers viral episomal DNA to host chromosome in latent phase.	[257]
			BGLF4	RanGAP1	Nuclear accmulation of RanGAP1 inhibits nuclear import of cNLS-bearing cargoes.	[258]
				FG-Nups (Nup62 and Nup153)	Phosphorylation of FG-Nups dilates NPC pores to facilitate nuclear import of non-NLS-bearing viral proteins.	
				Microtubule	Reorganizes nucleus shape to allow nuclear import of non- NLS-bearing viral proteins.	
			EGR1	Importin-7 (IPO7)	Negatively regulates importin-7 expression to a level that is optimal for EBV-transformed cell growth.	[261]
			EBV miRNA	Exportin-5 (XPO5)		
			EB2	Crm1	Boosts EB2-mediated gene expression and mediate unspliced lytic EBV mRNA export	[263]
				Nup214	EBV mRNA export for translation.	
			BFLF2 (Nuclear egress protein 1)	Ran, Importin-α7/β1, Importin-β2	Interacts with BFRF1 (nuclear egress protein 2) at inner nuclear membrane to form nuclear egress complex (NEC).	[265,267]
				Nxf1	Interacts with Nxf1 for BFLF2 nuclear export without RNA participation	[267]
			Tegument proteins (pBTRF1 and pBGFL3)	Crm1	Assembles with viral nucleocapsid for tegumentation in cytoplasm.	[271]

**Table 3:** Types of Nuclear Transport Inhibitors with Antiviral Activities.

Types of Inhibitors	Compound	Target	Target Viruses	Types of Studies	References
Host-specific nuclear import inhibitors	Ivermectin	Importin α	SARS-CoV-2	In vitro	[318]
			DENV1 (EDEN-1)	In vitro and in vivo	[279]
			DENV2 (EDEN-2)	In vitro and in vivo	
			DENV3 (EDEN-3)	In vitro and in vivo	
			DENV4 (EDEN-4)	In vitro and in vivo	
			DENV2 (NGC)	In vitro and in vivo	[282]
			ZIKAV (Asian/Cook Island/ 2014)	In vitro and in vivo	[282]
			CHIKV-Rluc	In vitro	[319]
			Adenovirus (HAdV-C5 and HAdV-B3)	In vitro and in vivo	[320]
			BK polyomavirus (BKPyV)	In vitro	[321]
	GW5074	Importin α	DENV2 (NGC)	In vitro	[281]
			ZIKAV (Asian/Cook Island/ 2014)	In vitro	[281]
	M9M	Importin β2	HSV-1	In vivo	[287]
Host-specific nuclear export inhibitors	Selinexor	Crm1	SARS-CoV-2	In vitro	[322]
	Verdinexor	Crm1	IAV	In vitro and in vivo	[323,324]
Virus-specific nuclear transport inhibitors	4-HRP	NS5	Flaviviruses (DENV, ZIKV)	In vitro and in vivo	[298,299,300]
	GS-6207 and GS-CA	Capsid Protein	HIV-1 and HIV-2	In vitro and in vivo	[301,302]
	Small-molecule inhibitor	PB1	IAV	In vitro and in vivo	[303]
	VPKS-2 and -5	VP24	EBOV	In vitro	[304]
	High-affinity macrocyclic peptide	VP24	EBOV	In vitro	[305]
	Type-I IFN	E1	HPV	In vitro	[307]
	Capsid Assembly Modulator	Core/Capsid Protein	HBV	In vitro and in vivo	[308,309,310,311,312,313,314]

**Table 4:** Clinical Trials of Various Nuclear Transport Inhibitors in Treating Viral Infections.

Drugs	Disease	Clinical Trial Identifier	Study Design	Phase	Dose and Duration	Main Outcomes and Measures	Results	References
Ivermectin	COVID-19	NCT04405843	Double-blind RCT: Placebo (n: 200) vs. Ivermectin (n: 200)	Phases 2 and 3	Oral Ivermectin 300μg/kg of body weight for 5 days	Time to resolution of symptoms within a 21-day follow-up period	Ivermectin did not significantly shorten time to resolution of symptoms	[325]
		NCT04392427	Non-RCT: Standard Care Therapy (n: 61) vs. Combined Antiviral Therapy (n: 69)	Phase 1	S.C.T: Paracetamol tablets (3 times/day), Zinc supplement (2 times/day), Azithromycin (case by case)	Viral clearance rate	Viral clearance rate was significantly higher in CAT compared with SCT group (CAT vs. SCT 58.1%: 0% on day 7; 73.1%: 13.7% on day 15)	[326]
					C.A.T: Nitazoxanide (500mg/6 h), Ribavirin 1200mg (400mg divided doses), Ivermectin (following weight schedules)			
		NCT04407130	Double-blind RCT: Placebo, Ivermectin, Ivermectin + Doxycycline (n:24/group)	Phase 2	Oral Ivermectin (12mg/day), Doxycycline (200mg on day 1, followed by 100mg/12hours) for 5 days	Viral clearance and remission of clinical symptoms (fever, cough)	Ivermectin monotherapy significantly enhanced viral clearance (Ivermectin vs. placebo: 9.7 days: 12.7 days).	[327]
							No significant difference between the placebo group and Ivermectin + Doxycycline group.	
							No significant improvement in clinical symptoms (fever, cough, sore throat)	
		NCT04390022	Double-blind RCT: Placebo vs. Ivermectin (n: 12/group)	Phase 2	Oral Ivermectin (400μg/kg) for 7 days	Proportion of patients with detectable SARS-CoV-2 RNA by PCR from nasopharynx- geal swab at day 7 post-treatment.	Ivermectin did not significantly reduce viral loads, but Ivermectin-treated patients recovered faster from hyposmia/ anosmia.	[335]
	Dengue Fever	NCT02045069	Two consecutive double-blind RCT: Placebo (n:103) vs. Ivermectin: (n:100)	Phase 2 then proceeded to Phase 3	Phase 2: 2 or 3 days of 400μg/kg/day Ivermectin. Phase 3: 3 days of 400/kg/day Ivermectin	Clinical progress and drug side effects	Ivermectin significantly improved NS1 antigenemia clearance time. The proportion of patients with detectable plasma NS1 was significantly lower in Ivermectin group compared to placebo. No significant difference in viremia clearance time between two groups	[329]
GS-6207 (Lenacapavir)	HIV	NCT03739866	Double-blind RCT: Placebo (n: 8) vs. GS-6207 (n: 32)	Phase 1	Four GS-6207 groups in dose escalation fashion: 20, 50, 150, 450 mg, daily single subcutaneous dose for 9 days	Virus clearance assessment, drug resistant assessment	Reduction in plasma HIV RNA in a dose-dependent fashion	[301]
							Resistant strain was found in one patient given 20mg GS-6207. Results showed a decrease in phenotypic susceptibility but no viral escape on day 9 of GS-6207 monotherapy.	
REBACIN^®^	HPV	-	Two independent, parallel RCTs: Placebo (n: 39) vs. REBACIN^®^ (n: 40)	-	REBACIN^®^ 0.5g per dose, every other day for 3 months except during the menstrual period	HPV viral clearance rate	Viral clearance rates: REBACIN^®^ 61.5 and 62.5% vs. Placebo 20.0 and 12.5%	[331]
NVR 3-778	HBV	NCT02112799 and NCT02401737	Phase 1a in NCT02112799 was to evaluate the safety of NVR3-778 in health volunteers. Phase 1b RCTs of both NCT02112799 and NCT02401737 focused on high viremic chronic HBV infected patients. Placebo (n:10) vs. Treatment (n: 63)	Phase 1a and 1b	7 treatment groups: 5 dose-escalation NVR 3-778 monotherapy groups; 1 combined group (NVR 3-778 + pegIFN); and 1 pegIFN monotherapy (placebo + pegIFN). 4 weeks treatment followed by 4 weeks post-treatment follow-up	Adverse effect assessment, virology assessment, drug resistance assessment, pharmacokinetic assessment.	Patients treated with more than 1200mg daily NVR 3-778 had lower serum HBV RNA and DNA. Synergistic effect in NVR 3-778 and pegIFN exerted strongest antiviral efficacy. No dose-related adverse effects reported.	[333]
					NVR 3-778 monotherapy doses: 100, 200, and 400mg once daily, 600mg and 1000mg twice daily; pegIFN monotherapy: 180μg/weekly			
					Combination therapy: NVR 3-778 600mg twice daily + pegIFN 180μg/weekly			
GLS4	HBV	CTR20160068	Double-blind RCT: Entecavir (control) vs. GLS4 + Ritonavir (n:8 per group)	Phase 1b	Control Entecavir 0.5mg; GLS4 120mg + Ritonavir 100mg; GLS4 240mg + Ritonavir 100mg for 28 days treatment	Adverse effect assessment, antiviral assessment, HBV serum markers assessment, and drug resistant assessment	120mg GLS4 was well tolerated and exerted antiviral activity in patients with chronic HBV infection	[334]
